# Muir-Torre Syndrome: Abdominal Sebaceous Carcinoma

**DOI:** 10.7759/cureus.33103

**Published:** 2022-12-29

**Authors:** An Bui, Shalini Shah, Nutan Winston, Ahmed Mahmoud

**Affiliations:** 1 Surgery, Riverside Community Hospital, Riverside, USA; 2 Anesthesiology, Riverside Community Hospital, Riverside, USA

**Keywords:** abdominal cystic mass, muir torre syndrome, muir-torre syndrome, sebaceous carcinoma, sebaceous gland

## Abstract

A 56-year-old male with a past medical history of arachnoid cysts and with two previous brain operations and a ventriculoperitoneal shunt presented with a large left lower quadrant (LLQ) abdominal mass for one year, growing rapidly for the past three months. He endorsed pus and blood leaking from ulceration on the lateral underside of the mass, and a section of the mass ruptured with blood and pus draining out on the day of admission. He denied any fevers, chills, pain, or numbness in the mass or any history of similar masses. Of note, the patient has an extensive familial history of cancer including colorectal cancer in his mother in her 30s. Computed tomography and biopsy of mass were concerning for malignancy, reporting a neoplasm in the dermis that grows in round and irregular lobules of cells along with the majority of the cells having oval nuclei with areas of sebaceous differentiation. Pathology showed sebaceous carcinoma with concern for Muir-Torre syndrome. The patient was discharged and instructed to follow up with oncology and gastroenterology.

## Introduction

Sebaceous carcinoma is a rare malignant tumor of the sebaceous gland which only occurs in 1-2 per 1,000,000 patients each year [1]. Eighty percent of sebaceous carcinoma cases are present in the integument of the head or neck, with half of these located in the periocular region [2]. Although most sebaceous carcinomas occur sporadically, in a patient under 60 years old with an extensive familial history of early-onset cancers suspicion should be raised for Muir-Torre syndrome (MTS), a variant of hereditary nonpolyposis colorectal cancer syndrome also known as Lynch syndrome [3]. MTS is characterized by sebaceous neoplasms, keratoacanthomas, and internal malignancy. The pathologic immunohistochemistry results from this patient’s mass show loss of nuclear expression of melanocyte-stimulating hormone (MSH) 2 and MSH6 indicating mismatch repair protein deficiency/high-level microsatellite instability raises our suspicion for a hereditary cancer syndrome such as MTS, and indicate germline testing and follow up with oncology.

## Case presentation

**Figure 1:**
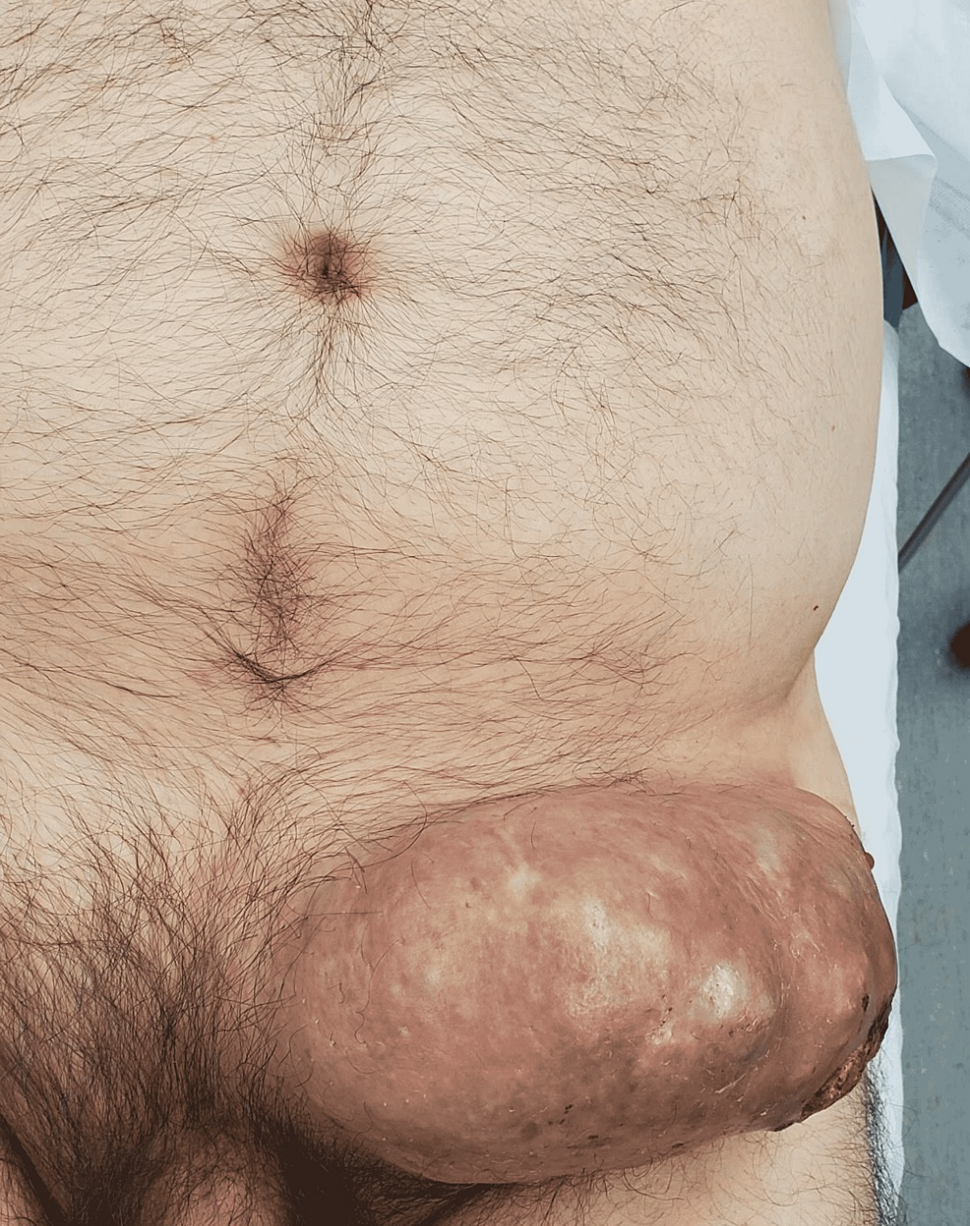
Abdominal wall mass with lateral underside ulceration pre-op

**Figure 2:**
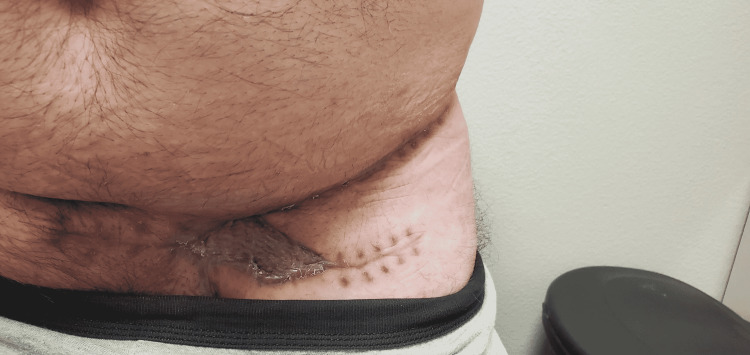
Surgical wound after mass excision with a portion left open pending pathology

The patient presented with abdominal pain with a large left lower quadrant abdominal wall mass for one year. The patient reported the mass began as an “ingrown hair” which resolved on its own, but returned and had been growing rapidly for the past three months, and had been leaking pus and blood from ulceration on the lateral underside of the mass (Figure 1). On the day of presentation, the patient reported a section of the mass ruptured and roughly 2 pints of blood and pus drained out which eventually subsided with pressure to the ulceration. He denied any pain or numbness in the mass or any history of similar masses. The patient had not been seen by a medical provider in five years and had never been seen by a dermatologist or oncologist. Of note, the patient has a family history of cancer with colon cancer, unspecified skin cancer, and lung cancer in his mother in her 30s. He reported several other family members diagnosed with unspecified cancers in their 30s or 40s. Vital signs include blood pressure of 133/85, heart rate of 69, temperature of 98.1, respiratory rate of 18, and saturation of 94% on room air. The complete blood count on presentation was an elevated white blood cell count of 11.4K/mm^3^. Computed tomography of the abdomen and pelvis showed a large multi-loculated fat-containing mass in the left lower abdominal wall measuring 16 cm x 9.5 cm x 3.2 cm concerning for possible fat-containing malignancy with superimposed infection possible. Images of mass and post-excisional surgical wounds were provided (Figure 2).

## Discussion

Sebaceous carcinoma is a rare tumor that a general surgeon may never encounter in their career, especially given only 20% are present outside of the head or neck with the most common site being the periocular region [2]. The most common site of metastasis in malignant sebaceous tumors is regional lymph nodes [2]. In patients diagnosed with extraocular sebaceous carcinomas, it is important to obtain a thorough immunohistochemical analysis of the tumor cells and to evaluate the patient for the possibility of MTS.

While the pathogenesis of extraocular sebaceous carcinomas is unclear, pathologic investigation often demonstrates mutations in common mismatch repair genes such as MutL Protein Homolog (MLH) 1 and MSH2, in addition to other common genes implicated in tumorigeneses such as tumor protein p53 and microsatellite instability [3]. These mutations may be somatic or inherited in a familial cancer syndrome. MTS, a variant of Lynch syndrome, is an autosomal dominant disorder characterized by at least one sebaceous skin tumor and at least one Lynch-related visceral cancer, commonly ovarian, endometrial, small bowel, colorectal, urinary tract, or biliary tract cancers [4]. Microsatellite instability is a strong indicator of mismatch repair gene mutation, which is a characteristic of Lynch syndromes. MTS is most commonly associated with a mutation in the mismatch repair gene MSH2 [5].

A useful tool for risk stratification in patients with extraocular sebaceous carcinoma is the Mayo MTS score, shown in Table 1.

**Table 1:** The Mayo MTS score for risk stratification MTS: Muir-Torre syndrome; HNPCC: hereditary non-polyposis colorectal cancer

Variable	Point(s)
Age at diagnosis of first sebaceous neoplasm	
≥60	0
˂60	1
Number of sebaceous neoplasms	
˂2	0
≥2	2
Personal history of HNPCC-related cancer	
No	0
Yes	1
Family history of any HNPCC-related cancer	
No	0
Yes	1

Lynch-related cancers as defined by this study included ovarian, endometrial, small bowel, colorectal, urinary tract, and biliary tract. The study showed MTS in 28/29 patients with a score of 3+, 12/20 patients with a score of 2, and 0/39 patients with scores of 1 or 0. It concluded that genetic testing for MTS is indicated in all patients with a score of 2 or greater [4].

## Conclusions

Our patient has a Mayo MTS score of 2 points, given that he is younger than 60 and has a family history of colorectal cancer in his mother. This indicates that he is at intermediate risk of MTS, and should have further genetic workup. Validating this finding are the pathology findings in his mass, which showed a lack of nuclear expression of MSH2 and MSH6. Although this could represent a somatic mutation, it is important to conduct germline testing given his family history. If our patient does have MTS he can be screened for other Lynch-related cancers.

Although sebaceous carcinoma located outside of the head and neck is exceedingly rare, it has been described in case reports multiple times before. Our case is significant not only for its rarity, but also for its possible connection to MTS given the pathology results and the strong familial cancer history.
